# Extracellular vesicles of *Bifidobacterium longum* reverse the acquired carboplatin resistance in ovarian cancer cells via p53 phosphorylation on Ser15

**DOI:** 10.1002/kjm2.12837

**Published:** 2024-04-22

**Authors:** Yun‐Long Fan, Jia‐Xi Jin, Jun Zhu, Hai‐Bo Ruan, Jin‐Qun Huang

**Affiliations:** ^1^ Departments of Gynaecology and Obstetrics The First People's Hospital of Wenling Wenling China

**Keywords:** chemoresistance, extracellular vesicles, invasion, microme, ovarian cancer

## Abstract

We previously found that the relative abundance of *Bifidobacterium* was increased after chemotherapy; however, the role of *Bifidobacterium longum* in chemotherapeutic drug resistance in ovarian cancer (OVC) remains unclear. This study aimed to understand the potential effects and mechanism of *B. longum* extracellular vesicles (*B. longum*‐EVs) on carboplatin (CBP) resistance in OVC. Eight normal and 11 ovarian tissues were collected and the expression of *B. longum* genomic DNA and its association with acquired CBP resistance in OVC patients was determined. After isolating EVs by ultracentrifugation from *B. longum* (ATCC 15707), CBP‐resistant A2780 cells were treated with PBS, CBP, *B. longum*‐EVs, or CBP + *B. longum*‐EVs, and subsequently analyzed by CCK‐8, Edu staining, Annexin V/PI double staining, wound healing, and Transwell assays to detect cell viability, proliferation, apoptosis, migration, and invasion, respectively. MRP1, ATP7A, ATP7B, and p53 expression as well as p53 phosphorylation were measured by western blot analysis. S15A mutation of p53 was assessed to examine the potential role of p53 Ser15 phosphorylation in CBP‐resistant OVC. *B. longum* levels were elevated and positively associated with CBP resistance in OVC patients. Only high concentrations of *B. longum*‐EVs attenuated A2780 cell proliferation, apoptosis, migration, and invasion. *B. longum*‐EVs exposure significantly enhanced the sensitivity of CBP‐resistant A2780 cells to CBP and decreased the expression of drug resistance‐related proteins. The effect of *B. longum*‐EVs on reversing CBP resistance was completely inhibited by S15A mutation of p53. *B. longum*‐EVs enhanced the sensitivity of OVC cells to CBP through p53 phosphorylation on Ser15.

## 1 INTRODUCTION

As the sixth most common female tumor, ovarian cancer (OVC) is a fatal gynecological malignancy, with approximately 314,000 new cases and more than 207,000 deaths reported in 2020 worldwide.^1^ Although improved surgery and combination chemotherapy have significantly improved the management of OVC over the past few decades; however, the cure rate remains low.^2^ Greater than 90% of patients with OVC can be cured by currently available treatment regimens if the cancer is limited to the ovaries at diagnosis^3^; however, most patients present with advanced‐stage disease, in which the cancer cells have metastasized beyond the primary tumor. These tumor cells are or tend to become resistant to front‐line therapies. Therefore, developing effective strategies to overcome chemoresistance is important for OVC management.

Recent evidence suggests that the microbiome is involved in the development of various cancers.^4^ During the last decade, studies have demonstrated that changes within the microbiome of the gastrointestinal tract and even the female reproductive tract may influence cellular processes, such as the cell cycle and apoptosis, and modify the host's hormone metabolism and immune response, thereby promoting a procarcinogenic environment.^5^ A previous report showed that the presence of oral *Aggregatibacter actinomycetemcomitans* and *Porphyromonas gingivalis* increases the risk of developing pancreatic cancer.^6^ Moreover, *Fusobacterium nucleatum* contributes to not only colorectal cancer development, but also malignant phenotypes of colorectal cancer including chemoresistance, metastasis, and recurrence.^7^, ^8^ In contrast, probiotics may play a role in inhibiting the occurrence and development of several cancers.^9^ The administration of *Lactobacillus plantarum* effectively reduces the incidence of dextran sulfate sodium‐induced colon cancer in mice.^10^ Zhang et al. showed the potential of *Lactobacillus salivarius* in the suppression of oral carcinogenesis.^11^ A multicenter study revealed that the habitual intake of *Lactobacillus casei* results in a reduction in the risk of bladder cancer.^12^ The antitumor effect of probiotics has gained interest in the field of cancer prevention and anticancer therapies; however, there are few studies describing a role of probiotics in OVC. Thus, it is of significance to conduct studies on the anticancer anticancer properties of specific probiotic strains and their underlying mechanisms in OVC.

Our previous study using clinical samples from OVC patients revealed that the relative abundance of *Bifidobacterium* increases following chemotherapy.^13^ We hypothesized that *Bifidobacterium* is involved in the anticancer activity of drugs against OVC. Bacteria can communicate with their host through direct contact and by secreting soluble products, such as extracellular vesicles (EVs). EVs are a heterogeneous group of small, lipid‐bound nanoparticles that are released from cells into the surrounding environment. They can transport cargo (DNA, RNA, and proteins) between cells as a form of intercellular communication.^14^ Bacterial EVs in the blood and body fluids may be involved in inflammation and mortality associated with several human disorders.^15^ Intraperitoneal injection of *Escherichia coli*‐derived EVs caused a sepsis‐like syndrome resulting in fatality in mice.^16^ In addition, several studies reported that EV‐based vaccines trigger protective immunity against pathogenic bacterial infections.^17^ To date, there are few studies examining how bacterial EVs can affect the occurrence and progression of cancer. Notably, the promising potential of some bacterial EVs in cancer immunotherapy was demonstrated in a recent study.^18^

Based on these findings, we focused on the effect and mechanism of EVs derived from *Bifidobacterium longum* on carboplatin (CBP) resistance in OVC. Our goal is to provide information regarding the potential of *B. longum*‐EVs to increase CBP sensitivity in drug‐resistant OVC patients.

## 2 MATERIALS AND METHODS

### 2.1 Clinical samples and data collection

A total of 19 tissue samples, including eight benign ovarian tumors and 11 OVC tissue samples were collected from patients who underwent surgical resection at our hospital. All OVC patients included in this study had received CBP chemotherapy, of which clinicopathological data were collected through the corresponding pathology records. Clinical sample and data collection were conducted after obtaining informed consent from the patients as well as approval from our hospital's Institutional Review Board (Approval no. KY‐2023‐1010‐01).

### 2.2 RNA extraction and qRT‐PCR

Microbial genomic DNA (gDNA) was extracted from the samples using the Qiagen RNeasy Kit based on the manufacturer's instructions, followed by RNA quantitation using a UV spectrophotometer. The PrimeScript RT Reagent Kit and the SYBR Premix Ex Taq II Kit were used to measure the expression of bacterial 16S rRNA genes for *B. longum* with the following primer set: forward, TTCCAGTTGATCGCATGGTC; reverse, GGGAAGCCGTATCTCTACGA. qRT‐PCR was done using the Applied Biosystems real‐time PCR system with the amplification program as previously described.^19^ The products were separated on a 1% agarose gel by electrophoresis prior to ethidium bromide staining.

### 2.3 *B. longum* culture and conditioned medium collection

The *B. longum* strain (ATCC No. 15707) was supplied by the American Type Culture Collection and cultured as recommended. *B. longum* was grown in deMan Rogosa Sharpe medium supplemented with l‐cysteine (0.05%, w/v) and cultured under anaerobic conditions at 37°C. *B. longum* supernatants were isolated from the conditioned media by centrifuging at 4000 × g for 10 min at 4°C and filtered through a 0.22‐μm membrane filter. The filtered media was stored at −80°C before EV isolation.

### 2.4 Extracellular vesicles of *B. longum* isolation and characterization

EV isolation was performed using ultracentrifugation. To obtain the *B. longum*‐EVs, the collected *B. longum*‐conditioned medium was subjected to ultracentrifugation at 200,000 × g for 60 min. The resulting pellets were resuspended in PBS and filtered through a 0.22‐μm syringe filter to obtain pure *B. longum*‐EVs.

The characterization of *B. longum*‐EVs, including morphology, number, and size distribution, was performed by transmission electron microscopy (TEM) and nanoparticle tracking analysis (NTA) by Nano‐ZS 90 dynamic light scattering. The remaining *B. longum*‐EVs were stored at −20°C until use.

### 2.5 Cell culture and establishment of CBP‐resistant OVC cells

The A2780 OVC cell line (Procell no. CL‐0013) was grown in DMEM media with 10% FBS at 37°C. CBP‐resistant A2780 (A2780‐CBP/R) cells were established as described by Viscarra et al.^20^ Briefly, the parental A2780 cells were exposed to intermittent and gradually increasing concentrations of CBP, with an initial concentration of 1 μM. Resistance was stable in A2780‐CBP/R cells when cultured in the presence of 10 μM CBP.

To confirm the successful establishment of A2780‐CBP/R cells, CBP resistance was analyzed by a CCK‐8 assay. Briefly, parental and A2780‐CBP/R cells were seeded into 96‐well plates and exposed to a series of concentrations (0, 5, 10, 25, 50, 100, and 200 μM) of CBP. CCK‐8 solution was added to each well after a 24‐h incubation. The cells were further incubated for 2 h and the optical density (OD) was measured for each well at 450 nm. The IC50 was determined by fitting the concentration versus cell viability curve using GraphPad Prism software. The resistance index (RI) was calculated as follows:

$$\mathrm{RI}=\frac{\;\mathrm{IC}50\;\text{value of}\;A2780-\mathrm{CBP}/R}{\mathrm{IC}50\;\text{value of}\;A2780}$$

### 2.6 Cell treatment

After reaching approximately 85% confluence, A2780 cells were exposed to low, medium, and high doses (1, 5, and 10 μg, respectively) of *B. longum*‐EVs to determine the effect of *B. longum*‐EVs on CBP sensitivity. A2780‐CBP/R cells were also treated with CBP, *B. longum*‐EVs, and the combination of the two to determine the role of *B. longum*‐EVs in reversing CBP resistance. Cells treated with PBS were designated the control. At the indicated time points, the treated cells were subjected to a series of experiments.

### 2.7 Cell Counting Kit‐8 (CCK‐8) assay

To measure cell viability, a CCK‐8 assay was performed in cells seeded in 96‐well plates, which were treated for 24, 48, and 72 h. Next, 10% CCK‐8 reagent (Dojindo) was added to each well and incubated for another 2 h and the OD of each well was measured at 450 nm using a microplate reader (Bio‐Rad, Hercules, CA, USA).

### 2.8 Edu staining

The Edu Staining Proliferation Kit was used to assess the proliferation of cells based on the manufacturer's instructions. Using a BX51 microscope, images were obtained to observe and calculate the number of Edu‐positive cells.

### 2.9 Annexin V/PI double staining

Treated cells were collected and washed twice with PBS. Then, 5 μL of Annexin V dye and 10 μL of propidium iodide dye solution (Sigma‐Aldrich, USA) were added and the cells were incubated for 30 min in the dark at 4°C. The apoptosis rate was evaluated by flow cytometry (FCM).

### 2.10 Wound healing

To evaluate cell migration, cells were seeded at 2 × 10^5^ cells/well into six‐well plates. A 200‐μL pipette tip was used to scratch the surface of the plate once a monolayer of cells had formed. The scratched cells were removed by three gentle rinses of PBS, and the remaining cells were treated and cultured in FBS‐free DMEM media for 24 h. A BX51 microscope was used to photograph the same position of the scratches at 0 and 24 h.

### 2.11 Transwell

Transwell chambers were pre‐coated with Matrigel, and the invasive ability of cells representing different treatments was evaluated. Briefly, cells were seeded into the upper chamber in 200 μL DMEM and treated. Simultaneously, 700 μL DMEM containing serum was added to the lower chamber. After 24 h, cells remaining in the upper chamber were removed, while the cells traversing the membranes to the lower chamber were fixed and stained with 0.1% crystal violet for 15 min. Using a BX51 microscope, the stained cells were photographed and counted in five random visual fields.

### 2.12 Western blot analysis

Total protein was extracted from cells with RIPA buffer on ice, separated by SDS‐PAGE, and electrophoretically transferred onto PVDF membranes. The membranes were subjected to blocking with 5% skim milk for 2 h. To measure the expression of proteins related to CBP resistance in cells with different treatments, the membranes were incubated overnight at 4°C with the following primary antibodies: MRP1 (#GTX116046, 1:1000; GeneTex), ATP7A (#MBS8517622, 1:1000; MyBioSource), ATP7B (#MBS8521393, 1:1000; MyBioSource), p53 (#ab26, Abcam; 1:800), p53 phosphorylated at Ser15 (p53 ser15) (#ab223868, 1:1500; Abcam), and β‐actin (#ab8226, 1:800; Abcam). The membranes were rinsed three times before incubation with HRP‐labeled secondary antibody (A0208, Beyotime). Finally, the protein bands were visualized and quantified using an ECL Substrate Kit and Image J software, respectively.

### 2.13 Site‐directed mutagenesis of p53 and cell transfection

Using the pcDNA3.1‐p53 plasmid, the QuickMutation™gene mutagenesis kit (Beyotime, D0206) was used to create a p53 Ser15 mutation (p53S15D) with the following primers: Forward: GTCGAGCCCCCTCTGGACCAGGAAACATTTTCA, Reverse: TGAAAATGTTTCCTGGTCCAGAGGGGGCTCGAC, by PCR mutation reactions and the DNA sequence was confirmed. A2780‐CBP/R cells were seeded into six‐well plates and grown to approximately 75% confluency, and transfected with plasmid using Lipofectamine 3000 (Invitrogen, USA), followed by a 24‐h incubation. The effect of p53 Ser15 mutation in A2780‐CBP/R cells was confirmed by western blot analysis.

### 2.14 Statistical analysis

All data are expressed as the mean ± standard error of the mean. A Student's *t*‐test was used for comparison between two groups. For multiple‐group conditions, a one‐way ANOVA was performed with Bonferroni's test. *P* < 0.05 was considered statistically significant.

## 3 RESULTS

### 3.1 *B. longum* abundance is low in OVC tissues and negatively correlated with CBP resistance

**FIGURE 1:**
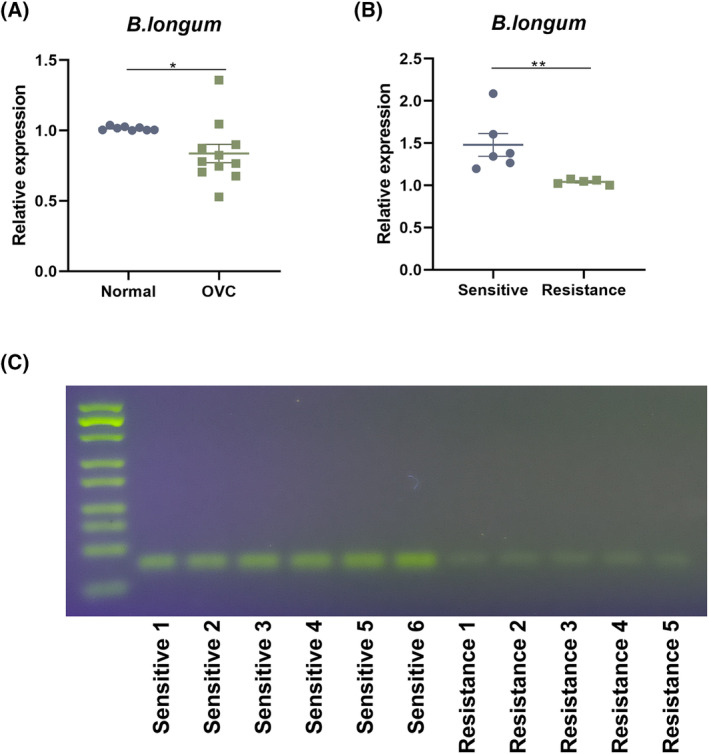
*Bifidobacterium longum* abundance is negatively correlated with carboplatin (CBP) resistance in ovarian cancer (OVC). Ovarian tissue samples were collected from patients including benign ovarian tumors (normal, *n* = 8) and OVC (*n* = 11, who received CBP chemotherapy), (A) the gDNA expression of *B. longum* in ovarian tissue samples from normal and OVC groups was measured by PCR. OVC patients were divided into sensitive (*n* = 6) and resistance (*n* = 5) groups based on whether they were resistant to CBP, (B) Northern blot of *B. longum* in sensitive and resistance groups. (C) The expression levels of *B. longum* in sensitive and resistance groups detected by qRT‐PCR. *P < 0.05 and **P < 0.01.

A significantly lower abundance of *B. longum* was observed in ovarian cancer tissues from patients compared with those with benign ovarian tumors (Figure 1A, *P* < 0.05). To examine the role of *B. longum* in OVC, the relationship of *B. longum* gDNA expression with patient clinical characteristics was analyzed. Among age, tumor size, pathological grade, CBP resistance, and metastasis, only CBP resistance exhibited a significant correlation with the abundance of *B. longum* in OVC patients (Table 1; *P* < 0.001). The expression level of *B. longum* gDNA in OVC tissues from CBP‐sensitive patients was significantly higher compared with that in CBP‐resistant patients (Figure 1B, C; *P* < 0.001). These data suggest the potential of *B. longum* to contribute to chemotherapeutic drug sensitivity in OVC patients.

**TABLE 1:** Association between *Bifidobacterium longum* gDNA expression and clinical characteristics.

	All patients	*B. Longum* gDNA		*p*‐value*
		Low expression	High expression	
Total number	11	6	5	
Age				0.819
<60	7	4	3	
≥60	4	2	2	
Median (range)	54 (39–73)			
Tumor size				0.3765
<5	5	2	3	
≥5	6	4	2	
Pathological grade				0.819
I–II	7	4	3	
III–IV	4	2	2	
Resistance				0.0057
Yes	5	5	0	
No	6	1	5	
Metastasis				0.3765
Yes	5	2	3	
No	6	4	2	

### 3.2 Effect of *B.* *longum*‐EVs on the proliferation, apoptosis, migration, and invasion of OVC cells

**FIGURE 2:**
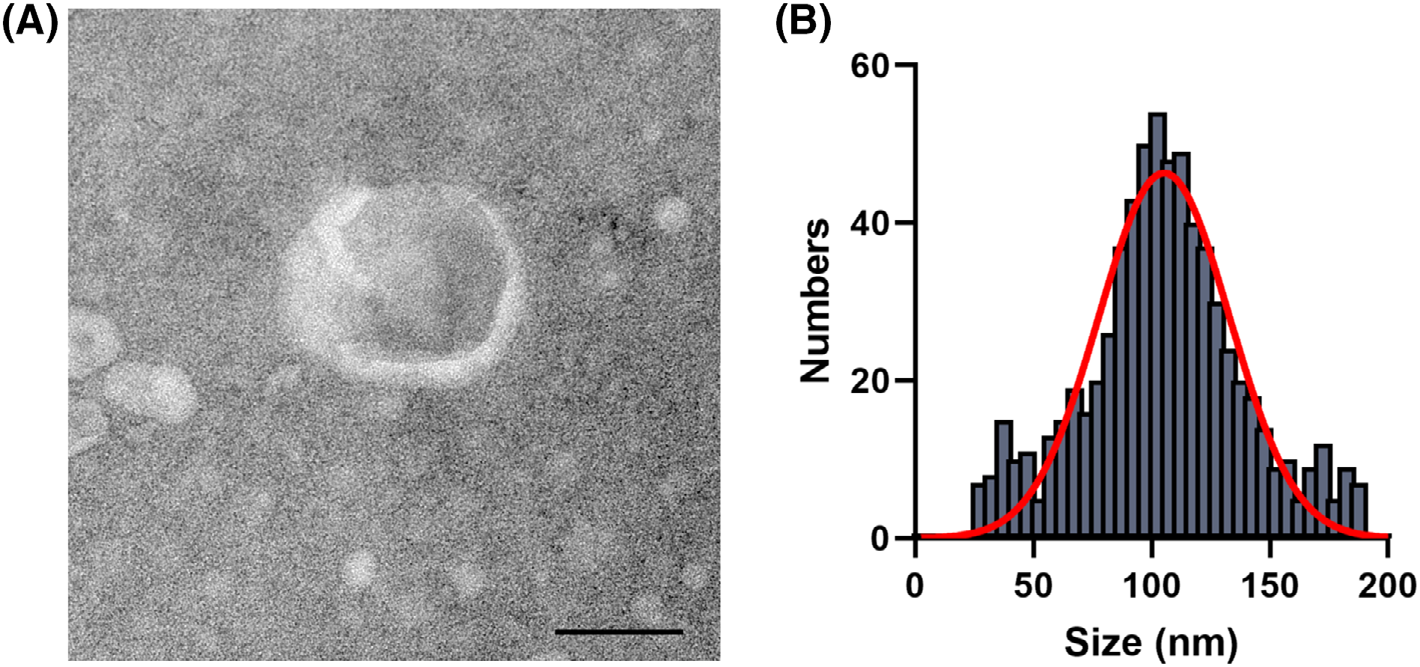
Characterization of *Bifidobacterium longum*‐EVs. *B. longum*‐EVs were isolated from the cultured supernatant of *the B. longum* strain (ATCC 15707) by ultracentrifugation. (A) Morphology of isolated *B. longum*‐EVs observed by TEM. (B) Size distribution of *B. longum*‐EVs analyzed by NTA.

Bacteria have a regulatory role in various diseases by secreting EVs. The effect of *B. longum*‐EVs on the proliferation, apoptosis, migration, and invasion of OVC cells was examined. Using a centrifugal ultrafiltration‐based method, *B. longum*‐EVs were isolated from culture supernatants of *B. longum* (ATCC 15707). TEM revealed that the isolated *B. longum*‐EVs with a round or oval membrane (Figure 2A). Meanwhile, the NTA results indicated that the peak diameter of the *B. longum*‐EVs was approximately 105 nm, of which the diameters ranged from 25 to 190 nm (Figure 2B). These results confirm the successful isolation of *B. longum*‐EVs.

**FIGURE 3:**
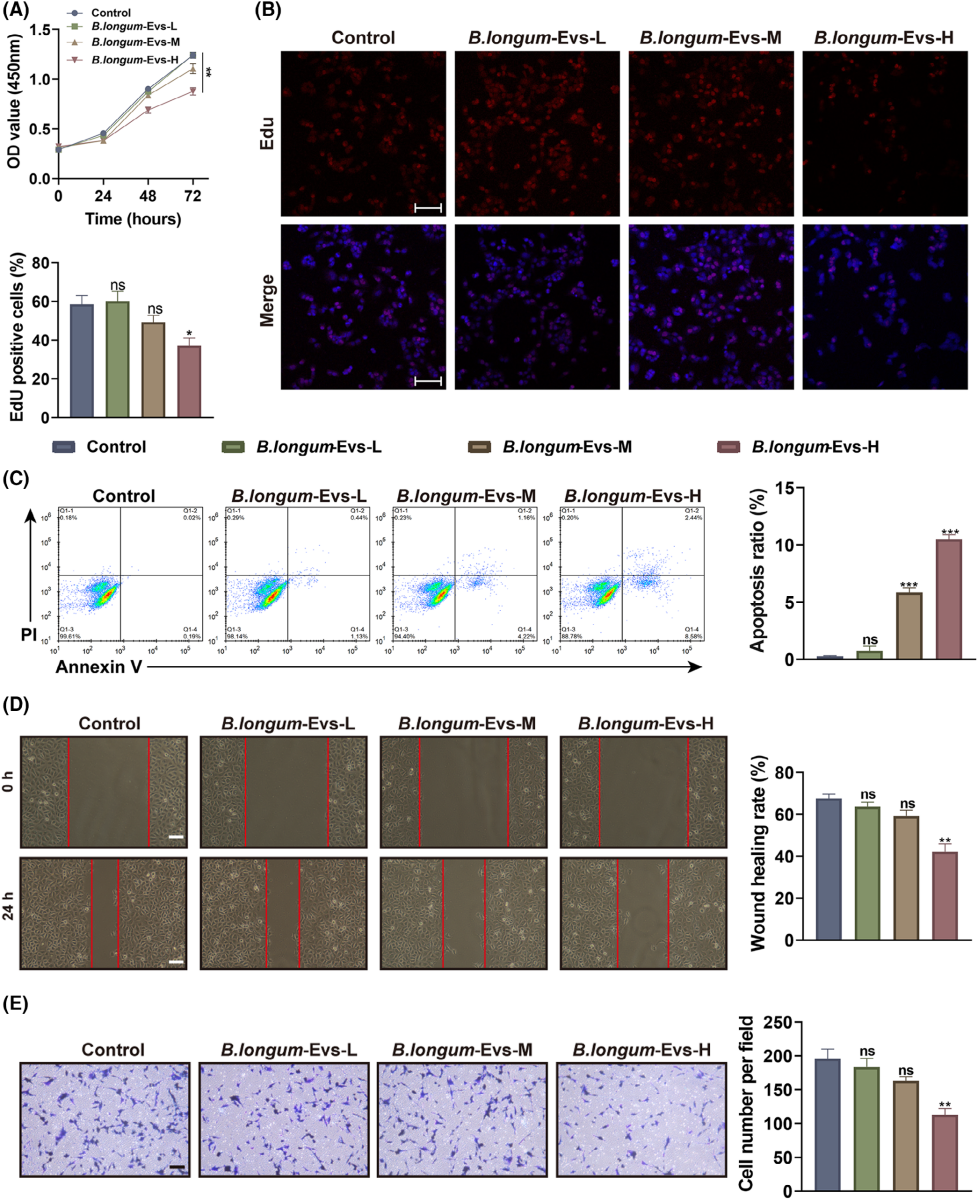
Effect of *Bifidobacterium longum*‐EVs on the proliferation, apoptosis, migration, and invasion of A2780 cells. A2780 cells were incubated with 1, 5, and 10 μg/mL of *B. longum*‐EVs (defined as *B. longum*‐EVs‐L, *B. longum*‐EVs‐M, and *B. longum*‐EVs‐H groups, respectively). (A) Cell viability was measured by the Cell Counting Kit‐8 (CCK‐8) after treatment for 24, 48, and 72 h. After a 24 h treatment, (B) cell proliferation, (C) apoptosis, (D) migration, and (E) invasion were detected by Edu staining, Annexin V‐FITC/PI double staining, wound healing, and Transwell assays, respectively. **P < 0.01 and ***P < 0.001, versus the control group.

A2780 cells were incubated with 1, 5, and 10 μg/mL of *B. longum*‐EVs (defined as *B. longum*‐EVs‐L, *B. longum*‐EVs‐M, and *B. longum*‐EVs‐H group, respectively) to determine whether EVs released by *B. longum* affect various cancer processes in recipient cells. After treatment with a diverse dose of *B. longum*‐EVs for 24, 48, and 72 h, only the *B. longum*‐EVs‐H group exhibited significantly lower cell viability compared with the control group (cells treated with PBS) (Figure 3A). Edu staining revealed that treating A2780 cells with a high dose of *B. longum*‐EVs resulted in a marked inhibition of cell proliferation (Figure 3B). FCM analysis revealed that *B. longum*‐EVs are capable of inducing apoptosis in A2780 cells at a concentration of 5 μg/mL (Figure 3C). Moreover, after the administration of *B. longum*‐EVs, the migration and invasion of A2780 cells were also markedly decreased at a high dose (Figure 3D, E). There was no significant difference between the control and *B. longum*‐EVs‐L groups in the proliferation, apoptosis, migration, and invasion of A2780 cells (Figure 3A–E). Taken together, *B. longum*‐EVs can inhibit the proliferation, anti‐apoptosis, migration, and invasion capacities of A2780 cells at a concentration of 10 μg/mL, but no effect was observed at 1 μg/mL.

### 3.3 *B.* *longum*‐EVs enhance the sensitivity of A2780‐CBP/R cells to CBP

The A2780‐CBP/R cell line was established using a moderate‐dose and intermittent treatment method. Using a linear fit, the IC50 values of A2780 and A2780‐CBP/R cells were calculated, which were 6.344 ± 1.039 and 19.846 ± 1.624 μM, respectively (Figure S1A). The RI of CBP in the A2780‐CBP/R cells was 3.179 ± 0.314 (Figure S1B).

**FIGURE 4:**
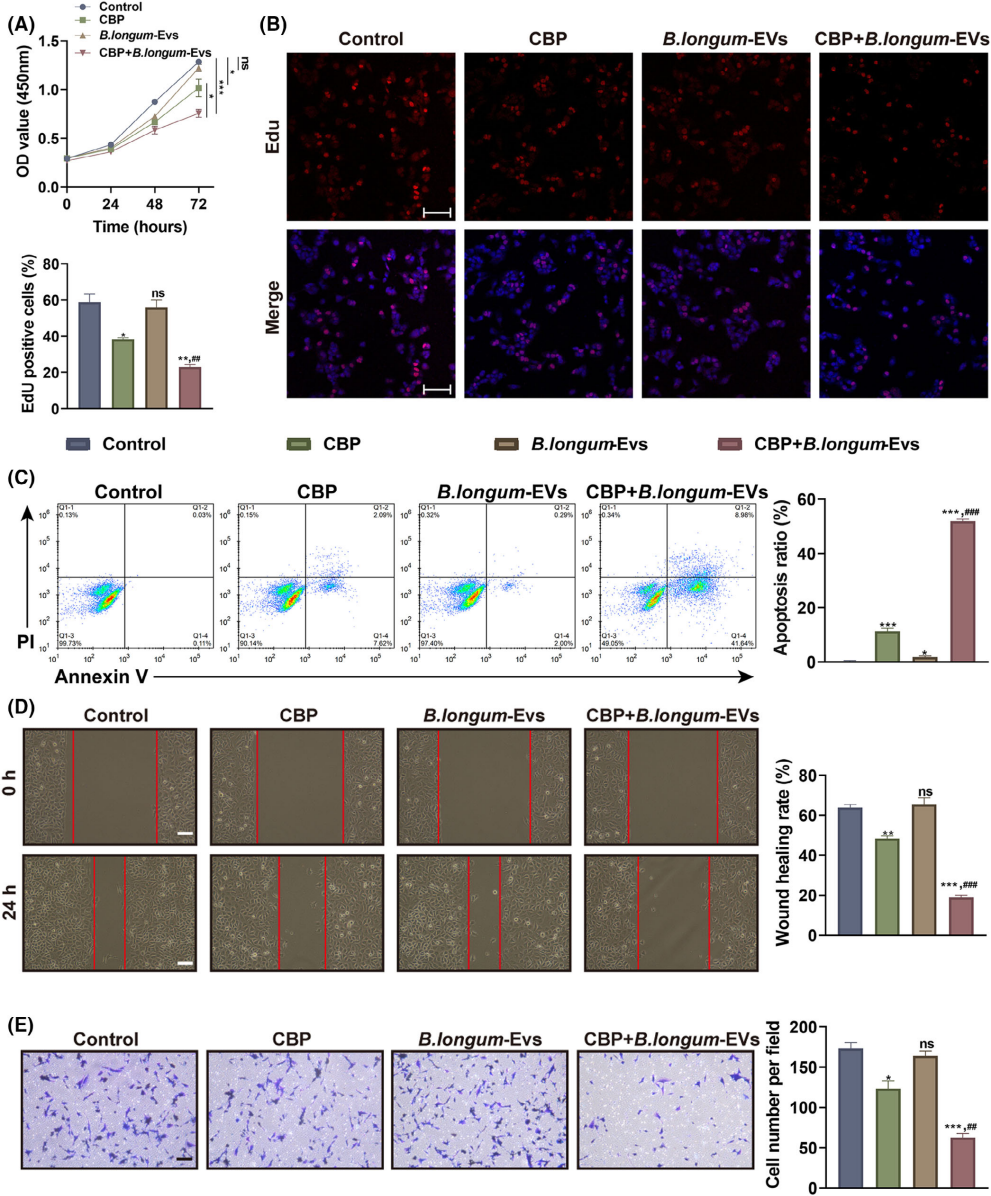
*Bifidobacterium longum*‐EVs enhances the sensitivity of A2780‐CBP/R cells to carboplatin (CBP). A2780‐CBP/R cells from different groups were treated with 10 μM of CBP, 1 μg/mL of *B. longum*‐EVs, or the combination, whereas cells treated with PBS were considered the control. (A) The cell viability was detected by CCK‐8 assay after treatment for 24, 48, and 72 h. After a 24‐h treatment, (B) cell proliferation, (C) apoptosis, (D) migration, and (E) invasion were detected by Edu staining, Annexin V‐FITC/PI double staining, wound healing, and Transwell assays, respectively. *P < 0.05, **P < 0.01, and ***P < 0.001, versus the control group; ^#*#*^ *P* < 0.01, and ^###^ *P* < 0.001, versus the CBP group.

After establishing the A2780‐CBP/R cell line, A2780‐CBP/R cells from different groups were respectively exposed to PBS, CBP (10 μM), *B. longum*‐EVs (1 μg/mL), and the combination of CBP with *B. longum*‐EVs, followed by a series of biological assays. Similar to the results in A2780 cells, 1 μg/mL of *B. longum*‐EVs did not significantly affect the cell proliferation, apoptosis, migration, as well as invasion of A2780‐CBP/R cells (Figure 4A–E). Treatment with 15 μM of CBP inhibited proliferation, migration, and invasion (Figure 4A, B, D, E), and induced apoptosis in A2780‐CBP/R cells (Figure 4C). Notably, the cytotoxicity of CBP in A2780‐CBP/R cells was markedly strengthened by *B. longum*‐EVs (Figure 4A–E), which suggests that *B. longum*‐EVs can effectively increase the sensitivity of A2780‐CBP/R cells to CBP.

### 3.4 *B.* *longum*‐EVs promote p53 Ser15 phosphorylation to increase p53 accumulation in A2780‐CBP/R cells

**FIGURE 5:**
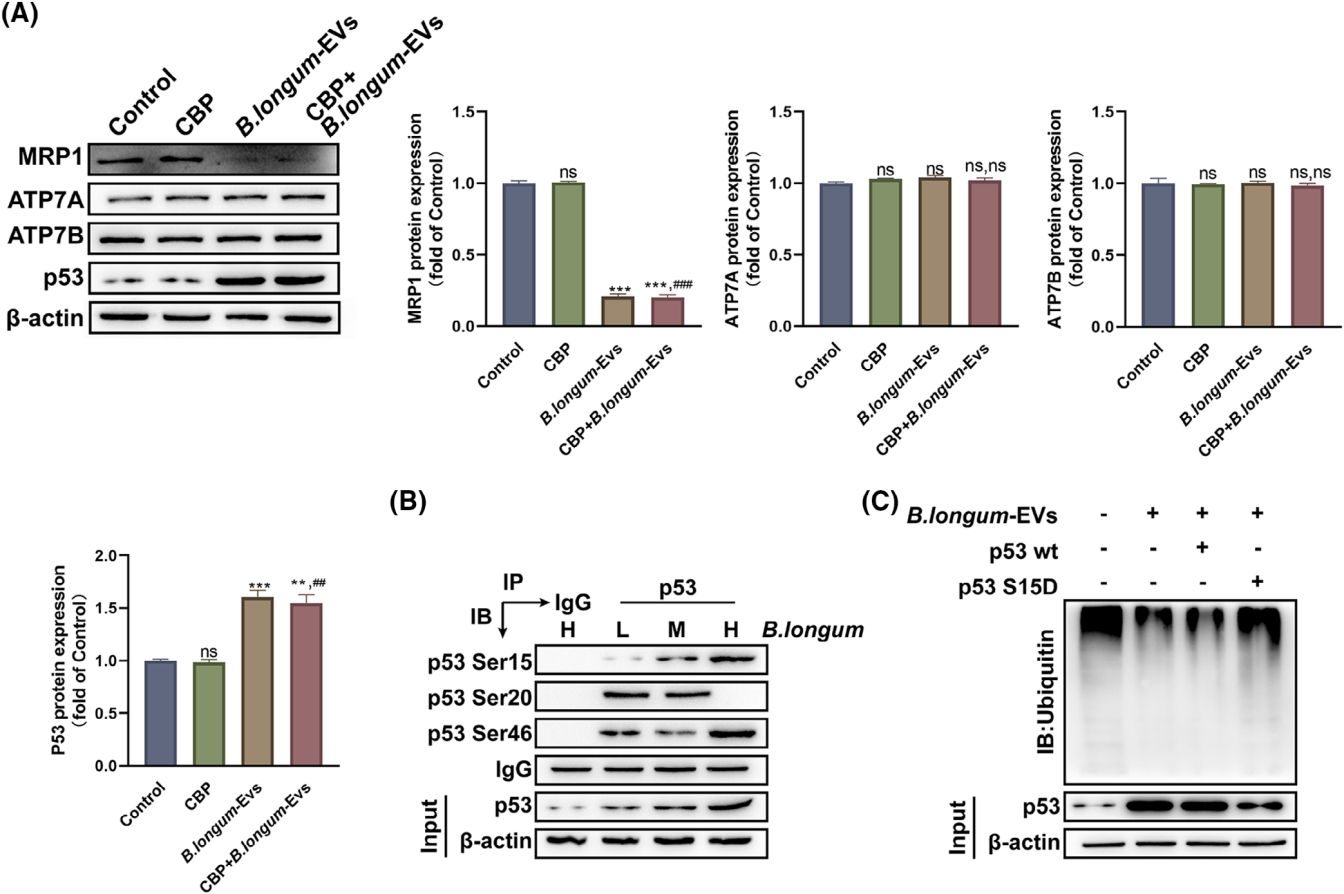
*Bifidobacterium longum*‐EVs promote p53 Ser15 phosphorylation to increase p53 accumulation in A2780‐CBP/R cells. (A) A2780‐CBP/R cells from different groups were treated with 10 μM carboplatin (CBP), 1 μg/mL of *B. longum*‐EVs, or the combination, whereas the cells treated with PBS were considered the control. After treatment for 24 h, cells from each group were harvested to analyze the expression levels of MRP1, ATP7A, ATP7B, and p53. (B) Extracts from A2780‐CBP/R cells from the *B. longum*‐EVs‐L, *B. longum*‐EVs‐M, and *B. longum*‐EVs‐H groups were immunoprecipitated with either control IgG or p53 antibodies, and the presence of p53 Ser15, Ser20, and Ser46 phosphorylation was determined by western blot analysis. (C) A2780‐CBP/R cells were transfected with p53S15D or p53wt or the combination with or without *B. longum*‐EVs. The cells were then treated with MG132 for an additional 6 h before collecting cell lysates for the ubiquitination assay. ***P < 0.001, versus the control group; ^#*#*^ *P* < 0.01, and ^###^ *P* < 0.001, versus the CBP group.

The mechanism underlying the role of *B. longum*‐EVs in reversing the CBP resistance of A2780‐CBP/R cells was examined. The expression of resistance‐related proteins that have been reported to contribute to CBP resistance (MRP1, ATP7A, ATP7B, p53, and MDM2) were different between A2780 and A2780‐CBP/R cells (Figure S1C). After treatment with *B. longum*‐EVs, a significant decrease in the expression of MRP1 was observed in A2780‐CBP/R cells, whereas the expression of ATP7A and ATP7B was unchanged (Figure 5A). Moreover, *B. longum*‐EVs markedly increased the expression of p53 total protein (Figure 5A).

Because the stability of the p53 protein is closely associated with its phosphorylation at several sites (15, 20, and 46), we determined whether *B. longum*‐EVs can affect the phosphorylation of p53, and its specific target site. The results indicated that the level of p53 Ser15 phosphorylation was affected by *B. longum*‐EVs in a dose‐dependent manner (Figure 5B). Therefore, we selected p53 Ser15 for further analysis. An in vivo ubiquitination assay revealed that *B. longum*‐EVs significantly decreased the ubiquitination of p53, suggesting that they can stabilize p53 expression by reducing its degradation (Figure 5C). After generating the p53 S15D mutant (p53S15D) by site‐directed mutagenesis, the *B. longum*‐EV‐mediated reduction of p53 ubiquitination in A2780‐CBP/R cells was clearly inhibited (Figure 5C). These results suggest that p53 Ser15 may be a target of *B. longum*‐EVs to promote p53 stability in A2780‐CBP/R cells.

### 3.5 *B.* *longum*‐EVs reversed CBP resistance by promoting p53 Ser15 phosphorylation in A2780‐CBP/R cells

**FIGURE 6:**
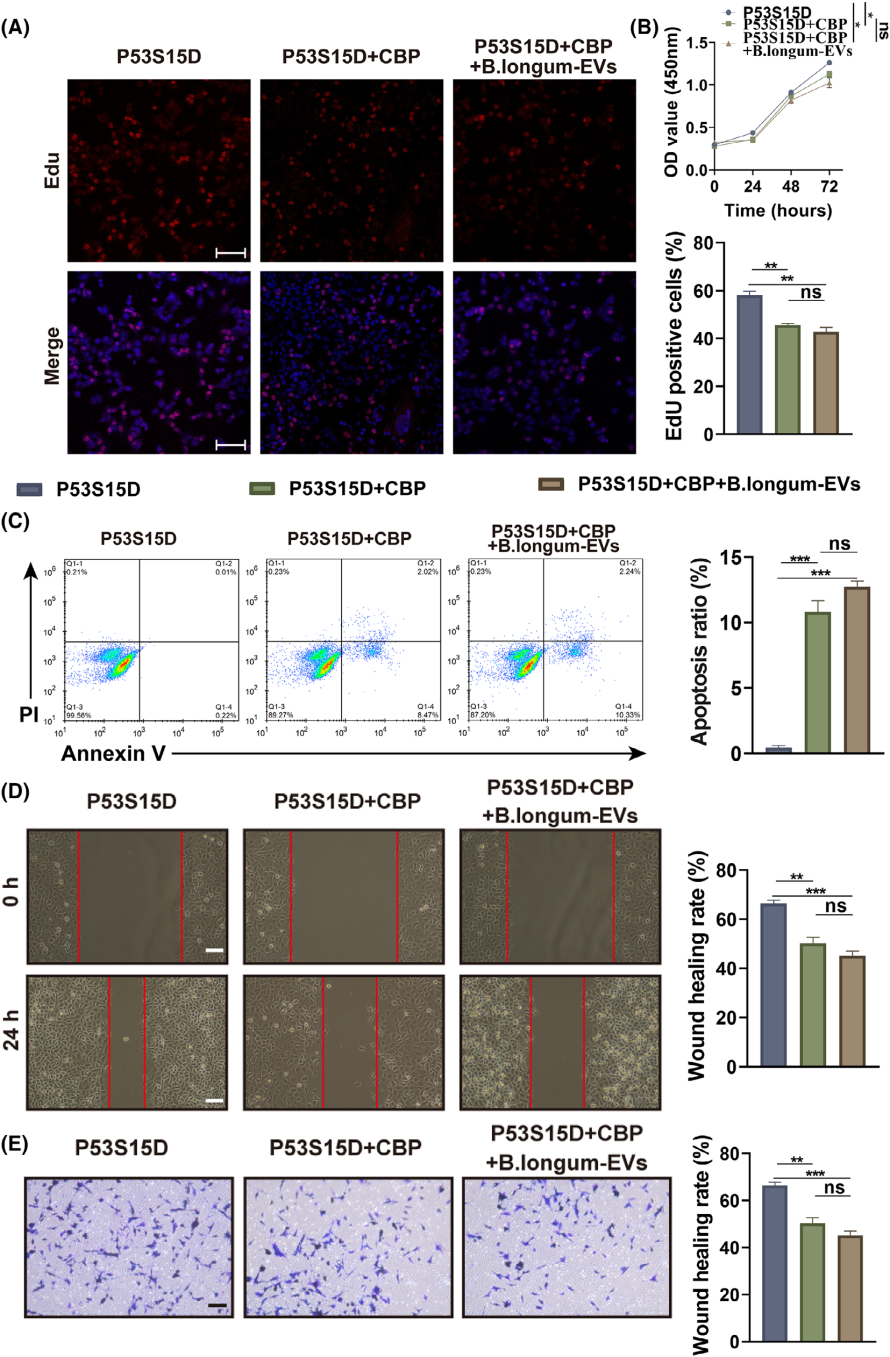
*Bifidobacterium longum*‐EVs reverse carboplatin (CBP) resistance by promoting p53 Ser15 phosphorylation in A2780‐CBP/R cells. The p53S15D mutant was generated in A2780‐CBP/R cells. A2780‐CBP/R cells with the p53S15A mutant were treated with CBP alone or combined with *B. longum‐*EVs, whereas the cells treated with PBS served as the control group. (A) Cell viability was measured by the CCK‐8 assay after treatment for 24, 48, and 72 h. After a 24‐h treatment, (B) cell proliferation, (C) apoptosis, (D) migration, and (E) invasion were detected by Edu staining, Annexin V‐FITC/PI double staining, wound healing, and Transwell assays, respectively. *P < 0.05, **P < 0.01, and ***P < 0.001.

To determine whether *B. longum*‐EVs reverse CBP resistance by promoting p53 Ser15 phosphorylation, p53S15D was generated in A2780‐CBP/R cells in a series of functional experiments. After the successful mutation of the Ser15 phosphorylation site on p53, the cells were treated with CBP alone or in combination with *B. longum‐*EVs. Cells treated with PBS served as a control group. CBP treatment did not effectively suppress cell proliferation, migration, or invasion (Figure 6A, B, D, E), but enhanced apoptosis in A2780‐CBP/R with p53S15A cells, (Figure 6C). After mutating Ser15 to Asp p53, there was no significant difference between the effect of CBP and CBP + *B. longum*‐EVs on A2780‐CBP/R cells (Figure 6A–E). This suggests that Ser15 mutant p53 nearly inhibited the ability of *B. longum*‐EVs to enhance the CBP sensitivity of A2780‐CBP/R cells.

## 4 DISCUSSION

Over the last decade, the advent of metagenomic sequencing has vastly enriched our knowledge of the diversity and abundance of microbes in the human body,^21^ whereas the vital role of the microbiome in tumor development has been demonstrated.^22^ Thus, the theory of “sterile” fallopian tubes and ovaries has encountered increased opposition.^23^ A recent study revealed that bacterial composition was significantly different between cancerous and noncancerous ovarian tissues.^24^ Our previous study indicated that the relative abundance of *Bifidobacterium* was increased after chemotherapy; however, whether *Bifidobacterium* contributes to the efficacy of chemotherapy remains to be elucidated. As an anaerobic probiotic, *Bifidobacterium* primarily consists of *B. longum*, *B. breve*, and *B. infantis*, which were reported to reduce the risk of neonatal necrotizing enterocolitis.^25^ Supplementation with *Bifidobacterium* also influences immunotherapy and significantly improves antitumor immunity in a mouse cancer model.^26^ A previous study showed that the administration of *B. longum* has therapeutic potential in murine colorectal cancer models through its modulation of tumor suppressor microRNAs.^27^ In this study, we identified *B. longum*‐EVs that have minimal effect on cell proliferation, apoptosis, migration, and invasion, but reverse the CBP resistance of OVC cells, and further uncovered the underlying molecular mechanisms.

In the present study, we found that the expression of *B. longum* in clinical samples from patients with OVC was significantly lower compared with patients with benign ovarian tumors, and negatively correlated with CBP resistance. These data suggest that *B. longum* inhibits tumor progression and the development of CBP resistance. A role for bacterial EVs in inflammation and mortality has been demonstrated in various human diseases.^28^, ^29^ Thus, we isolated EVs from the *B. longum* strain and examined their role in the malignant phenotype of OVC cells. Surprisingly, we found that a low concentration of *B. longum*‐EVs had no effect on the cell proliferation, apoptosis, migration, or invasion of A2780 cells, but effectively reversed the CBP resistance of A2780‐CBP/R cells.

As a first‐line chemotherapeutic drug for OVC, CBP exerts broad antitumor activity by facilitating DNA damage and mitochondrial apoptosis. Drug resistance is a major obstacle in the successful treatment of OVC. Many molecular mechanisms responsible for CBP resistance have been elucidated over the last decade.^30^ Based on the literature, there are primarily three molecular mechanisms involved in CBP resistance, including enhanced DNA repair, increased drug inactivation, and reduced cellular drug accumulation. As a widely reported multidrug resistance protein, MRP1 effluxes a variety of cytotoxic compounds from tumor cells and protects tumors from the effects of chemotherapeutic agents.^31^ In addition, copper‐transporting ATPases, such as ATP7A and ATP7B, are responsible for the excretion of platinum drugs, which also contribute to the CBP resistance phenotype.^32^ Our results indicated that these CBP resistance‐related proteins were all down‐regulated by *B. longum*‐EVs, supporting a role for *B. longum*‐EVs in reversing CBP resistance.

It is widely accepted that p53, a tumor suppressor, plays an important role in chemotherapy.^33^ In various tumor cell types, p53 was found to be inactive because of its enhanced degradation, even though it was intact.^34^ Increased evidence suggests that acquired p53 inactivation renders tumor cells resistant to chemotherapeutic drugs.^35^, ^36^ Several sites in p53 could be readily phosphorylated following DNA damage, whereas the phosphorylation of Ser15 and Ser20 are the most important events for the anti‐proliferative and apoptotic functions of p53.^37^, ^38^ A previous study demonstrated that p53 phosphorylation at Ser15 increased the ability of p53 to aggravate cisplatin‐induced apoptosis in OVC.^39^ In the present study, we found that interference of *B. longum*‐EVs resulted in a significant increase in p53 phosphorylation at Ser15. More importantly, in the presence of the S15A mutant of p53, the sensitivity of A2780‐CBP/R cells to CBP by *B. longum*‐EVs was abolished. This suggests that *B. longum*‐EVs function by promoting p53 phosphorylation at Ser15.

In the present study, the effects and underlying mechanisms of *B. longum*‐EVs on CBP resistance were identified for the first time. It should be acknowledged, however, some limitations exist in our study. First, the sample size of the tumor tissues used for *B. longum* gDNA expression analysis was limited. A larger sample size is needed to verify our findings in the future. Second, in vivo assays will be required to increase the reliability of the results of our in vitro experiments. Finally, future studies should focus on the proteomic and metabolic characterization of *B. longum*‐EVs to better understand their biological significance in CBP resistance.

In conclusion, we examined the role of *B. longum*‐EVs in OVC. The results indicated for the first time that *B. longum* is present at lower levels in OVC tumors relative to benign ovarian tumor tissue and down‐regulated in CBP‐resistant OVC. Moreover, our data showed that a low dosage (1 μg/mL) of *B. longum*‐EVs had no effect on the proliferative, anti‐apoptotic, migratory, and invasive capacities of A2780 cells, but significantly re‐sensitized A2780‐CBP/R cells to CBP by promoting p53 phosphorylation at Ser15. Our findings provide novel insight into the potential role of *B. longum*‐EVs in overcoming CBP resistance in OVC and hold promise for the future treatment of drug‐resistant OVC.

## CONFLICT OF INTEREST STATEMENT

The authors declare no conflicts of interest.

## Supporting information


**Figure S1.** Establishment of A2780‐CBP/R cells. The A2780‐CBP/R cell line was established using a moderate‐dose and intermittent treatment method.
