# Supplementary figures and images for: Extracellular vesicles of Bifidobacterium longum reverse the acquired carboplatin resistance in ovarian cancer cells via p53 phosphorylation on Ser15
Source: Kaohsiung J Med Sci. 2024 Apr 22;40(6):530–41. doi: 10.1002/kjm2.12837 (PMC11895628; doi:10.1002/kjm2.12837)

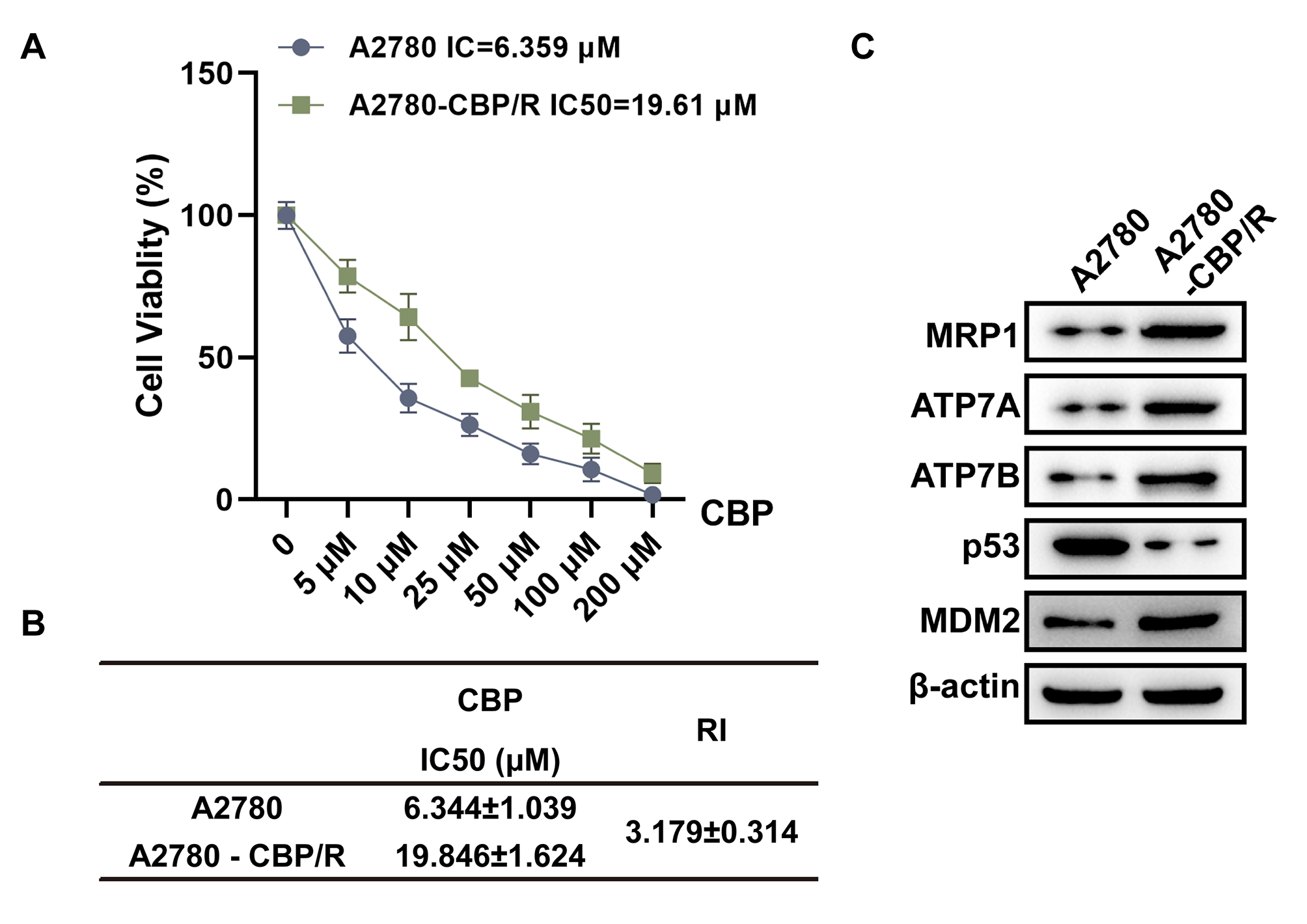

Supplement: Supplementary file 1 — Figure S1. Establishment of A2780‐CBP/R cells. The A2780‐CBP/R cell line was established using a moderate‐dose and intermittent treatment method. [file KJM2-40-530-s001.tif]
